# Determinants of loss to follow-up among HIV positive patients receiving antiretroviral therapy in a test and treat setting: A retrospective cohort study in Masaka, Uganda

**DOI:** 10.1371/journal.pone.0217606

**Published:** 2020-04-07

**Authors:** Julius Kiwanuka, Jacinta Mukulu Waila, Methuselah Muhindo Kahungu, Jonathan Kitonsa, Noah Kiwanuka

**Affiliations:** 1 AIDS Healthcare Foundation, Kampala, Uganda; 2 Department of Epidemiology and Biostatistics, School of Public Health, Makerere University Kampala, Uganda; 3 Medical Research Council / Uganda Virus Research Institute & London School of Hygiene and Tropical Medicine Uganda Research Unit, Entebbe, Uganda; CIRCB - Chantal BIYA International Reference Centre for research on HIV/AIDS prevention and management, CAMEROON

## Abstract

**Background:**

Retaining patients starting antiretroviral therapy (ART) and ensuring good adherence remain cornerstone of long-term viral suppression. In this era of test and treat (T&T) policy, ensuring that patients starting ART remain connected to HIV clinics is key to achieve the UNAIDS 90-90-90 targets. Currently, limited studies have evaluated the effect of early ART initiation on loss to follow up in a routine health care delivery setting. We studied the cumulative incidence, incidence rate of loss to follow up (LTFU), and factors associated with LTFU in a primary healthcare clinic that has practiced T&T since 2012.

**Methods:**

We retrospectively analyzed extracted routine program data on patients who started ART from January 2012 to 4^th^ July 2016. We defined LTFU as failure of a patient to return to the HIV clinic for at least 90 days from the date of their last appointment. We calculated cumulative incidence, incidence rate and fitted a multivariable Cox proportion hazards regression model to determine factors associated with LTFU.

**Results:**

Of the 7,553 patients included in our sample, 3,231 (42.8%) started ART within seven days following HIV diagnosis. There were 1,180 cases of LTFU observed over 15,807.7 person years at risk. The overall incidence rate (IR) of LTFU was 7.5 (95% CI, 7.1–7.9) per 100 person years of observation (pyo). Cumulative incidence of LTFU increased with duration of follow up from 8.9% (95% CI, 8.2–9.6%) at 6 months to 20.2% (95% CI, 19.0–21.4%) at 48 months. Predictors of elevated risk of LTFU were: starting ART within 7 days following HIV diagnosis ((aHR) = 1.69, 95% CI, 1.50–1.91), lack of a telephone set (aHR = 1.52, 95% CI, 1.35–1.71), CD4 cell count of 200–350μ/ml (aHR = 1.21, 95% CI, 1.01–1.45) and baseline WHO clinical stage 3 or 4 (aHR = 1.35, 95% CI, 1.10–1.65). Factors associated with a reduced risk of LTFU were: baseline age ≥25 years (aHR ranging from 0.62, 95% CI, 0.47–0.81 for age group 25–29 years to 0.24, 95% CI, 0.13–0.44 for age group ≥50 years), at least primary education level (aHR ranging from aHR = 0.77, 95% CI, 0.62–0.94 for primary education level to 0.50, 95% CI, 0.34–0.75 for post-secondary education level), and having a BMI ≥ 30 (aHR = 0.28, 95% CI, 0.15–0.51).

**Conclusion:**

The risk of loss to follow up increased with time and was higher among patients who started ART within seven days following HIV diagnosis, higher among patients without a telephone set, lower among patients aged ≥ 25 years, lower among patients with at least primary education and lower among patients with BMI of ≥ 30. In this era of T&T, it will be important for HIV programs to initiate and continue enhanced therapeutic education programs that target high risk groups, as well as leveraging on mHealth to improve patients’ retention on ART throughout the cascade of care.

## Introduction

By the end of 2017, the World Health Organization (WHO) estimated that globally about 36.9 million people were living with HIV (PLHIV) and 1.8 million new infections occurred that year; over two thirds of the new infections were in Sub Saharan Africa (SSA) with about 50,000 in Uganda. During the same year, about 21.7 million (~59%) of the PLHIV patients received antiretroviral therapy (ART) [1]. To accelerate epidemic control, the United Nations Joint Program on HIV/AIDS (UNAIDS) launched the 90-90-90 campaign in 2014. One of the targets was to achieve viral suppression among 90% of patients receiving ART by 2020 [2]. The other targets were ensuring that 90% of the people living with HIV know their HIV status and 90% of those who are diagnosed with HIV infection receive sustained ART. Treatment as prevention (TasP) studies have demonstrated effectiveness of ART in preventing new HIV infections [3–6]; therefore, increasing ART coverage has public health benefits including reduction of new HIV infections through reduced community viral loads. After two years of implementing the 2016 WHO and Uganda ministry of health (MoH) ART guidelines (T&T) [7,8], there was a 32.5% increase in the number of patients receiving ART from about 890,000 in June 2016 to close to 1.2 million by June 2018 [9,10]. Whereas achievement of the second and third UNAIDS targets demands timeliness in ART initiation, it is important that patients remain connected to the health care system for periodic drug refills and running of monitoring tests. Systematic reviews of studies on the rapidly expanding ART programs in SSA illustrated that about 60–65% of patients were retained in HIV care at 2 to 3 years after starting ART [11,12]. A study done in Uganda among patients who started ART with a CD4 cell count > 350 cells/ml found that at 2.5 years, 20% of patients were lost to follow up (LTFU) [13]. Similarly, the 2018 Uganda HIV/AIDS country progress report indicated suboptimal retention amongst ART naïve patients, with about 20% LTFU at 12 months [10]. Therefore, in settings where patients start ART immediately following a positive HIV test, there is the possibility that they may not return to the HIV clinics thereby offsetting the benefits associated with immediate initiation of treatment. Experiences from prevention of mother to child transmission (PMTCT) programs where immediate ART initiation has widely been practiced, among HIV infected mothers, indicate sub-optimal levels of ART adherence and retention of mothers in care, with majority being LTFU [14,15]. LTFU has been associated with drug resistance, and comparatively poor long-term treatment outcomes, including mortality [16]. In this era of T&T, it is important that data characterizing retention of patients is available, especially in routine health care delivery settings. However, most of the data currently available are derived from implementation of test-and-treat in research settings [17–19]. In this study, we set out to study the cumulative incidence, incidence rate of loss to follow up, and factors associated with loss to follow up in a primary healthcare clinic that had been implementing the T&T policy since 2012.

## Methodology

### Study design

This was a retrospective cohort study utilizing data collected on patients who were diagnosed with HIV and enrolled into the HIV care program from January 2012 to 4^th^ July 2016 at the Uganda Cares clinic located within the Masaka regional referral hospital. A patient’s ART initiation date defined the beginning of follow up (time zero) and follow up period was from 01^st^ January 2012 to 31^st^ December 2016. Patients with documented transfer out status contributed follow up time up to the date of transfer out. Patients’ follow up ended if they died, transferred out to another HIV service delivery point, were LTFU or censored on 31^st^ December 2016.

### Study site and settings

The Uganda Cares clinic serves as the main HIV outpatient department (OPD) clinic for Masaka regional referral hospital. The total catchment population for MRRH currently exceeds 2,000,000 people (according to the Uganda population and housing census-UPHC, 2014), distributed in almost ten districts. The HIV clinic runs five days a week and by the end of 2016, more than 13,000 clients were active in care, with more than **86%** starting ART. The clinic serves patients of all characteristics including sex workers from the various hot spots of the Kampala-Masaka-Mbarara high-way and from neighboring fishing communities.

### HIV testing, linkage to care and initiation of ART in the study setting

At the beginning of 2012, provider initiated counseling and testing (PITC) was scaled up in MRRH. At the same time, voluntary counseling and testing (VCT) as well as home based HIV Counseling and Testing (HBHCT) outreaches were scaled up in the nearby villages. A serial HIV testing algorithm was used during the study period. For HIV screening, one of either Determine^™^ HIV-1/2 (Alere Medical Company Limited, Chiba, Japan) or INSTI ® HIV-1/2 antibody test (Biolytical laboratories, Richmond, Canada) was used depending on availability. Stat-Pak^®^ Dipstick (Chembio Diagnostic Systems, Medford, NY—USA) was used as a confirmatory test and Uni-Gold^™^ HIV (Trinity Biotech, Bray, Ireland) was used for tie breaking. Clients diagnosed with HIV within the hospital were enrolled into HIV care and encouraged to start ART immediately or shortly after. During this period, a stand-alone desk (focal desk at the HIV clinic) was set up to fast track treatment initiation. Patients diagnosed with HIV at the outreach sites were referred to this focal desk by trained counselors, to further aid and expedite ART initiation. Although the clinic begun piloting a T&T strategy at the beginning of 2012, it is important to note that this strategy wasn’t a true manifestation of T&T as illustrated by the treatment as prevention (TasP) group, but rather a process where the ART initiation process was expedited, with the preparatory counseling phase taking a maximum of one week. Under this T&T strategy, point of care CD4 cell count and TB assessment were done within a week to further determine ART eligibility. However, patients who declined ART immediately or within seven days following HIV diagnosis, were initiated on ART at a time of their convenience with a similar array of services as their counterparts who started ART immediately or shortly after. Before June 2013, the MoH policy to start ART was based on CD4 cell ≤350 cells/ml or WHO clinical stages 3 or 4 [20]. From June 2013, HIV treatment guidelines have changed from starting ART based on CD4 cell count ≤500 cells/ml, WHO clinical stages 3 or 4 [21] to “treat all” regardless of CD4 cell count or WHO clinical stage [7,8].

### Study participants

We included all patients aged ≥18 years, tested HIV positive, who started ART anytime between 01^st^ January 2012 and 4^th^ July 2016, and were to be followed up at Masaka regional referral hospital. We excluded patients who were transferred in while on ART from other HIV clinics because we were not able to confirm their HIV test results and ART initiation dates with certainty, and patients with prior ART history (for example those that had ever used post exposure prophylaxis (PEP) since we could not ascertain the period when they were on medication).

### Variables, data sources and measurement

The primary outcome was loss to follow up defined as failure of the client to show up at the Masaka clinic for at least 90 days from the date of their last scheduled appointment [22] taking 31^st^ December 2016 as the reference date. We determined loss to follow up by comparing a patients’ most recent scheduled return visit date recorded into the electronic database with the reference date (31^st^ December 2016). The primary exposure (mode of treatment) was whether or not treatment was initiated within seven days following HIV diagnosis. Other extraneous variables included; patients’ sex, age at ART initiation (determined by subtracting date of birth from the date of initiating ART), level of education, marital status, baseline CD4 cell count, baseline WHO stage, TB status at enrollment, ownership of a telephone set, and baseline body mass index (BMI) calculated using the baseline weight and height according to the formula BMI = weight/height (m)^2^. We initially developed a data extraction checklist and electronically retrieved a sample of records with the site data manager. We compared data in this pilot sample with the patients’ files to ascertain the quality of data in the electronic database before we extracted the entire data set. Data were extracted in a Microsoft excel spread sheet, and cleaned. Where information was missing in the electronic database, the site data manager and two investigators retrieved patients’ charts (source documents) and corrected the gaps, helped by a team of expert patients. For completely missing records, we employed multiple imputation by chained equation methods and we used Stata® version 13 for analysis.

### Statistical methods

We summarized patients’ characteristics by medians (interquartile range) or mean (standard deviation) for continuous variables depending on the distribution, and percentages for categorical variables. Comparison of continuous and categorical baseline characteristics was done by using t-tests, Mann Whitney tests and chi-square or Fisher exact tests. We analysed data at bivariate level to estimate crude estimates or predictors of associations and multivariable cox regression modelling to estimate adjusted predictors of time to loss to follow up. Multivariable model building involved a stepwise approach. We included variables with a p value of <0.2 at bivariate and dropped each turning out with a P>0.05 at multivariable model building. Insignificant variables at this stage but highlighted in previous literature as significantly associated with LTFU were included in the final model. The proportion hazards (PH) assumption was evaluated for each of the variable included in the final model. Kaplan Meier survival curves were used to determine patients LTFU. In all statistical tests, a 5% level of significance was assumed.

### Ethical considerations

This study was retrospective and patients’ consent could not be obtained. This was acknowledged and waived by the Makerere University School of Public Health Higher Degrees Research Committee which granted study clearance. We also sought approval from the management of the HIV clinic, to allow us have access to patients’ data. Program data routinely collected and entered into an electronic records management system (OpenMRS) was extracted without patients’ direct identifying information. These patients’ data have been used solely for this current study, and cannot therefore be made publicly available.

## Results

### Baseline characteristics

**Fig 1:**
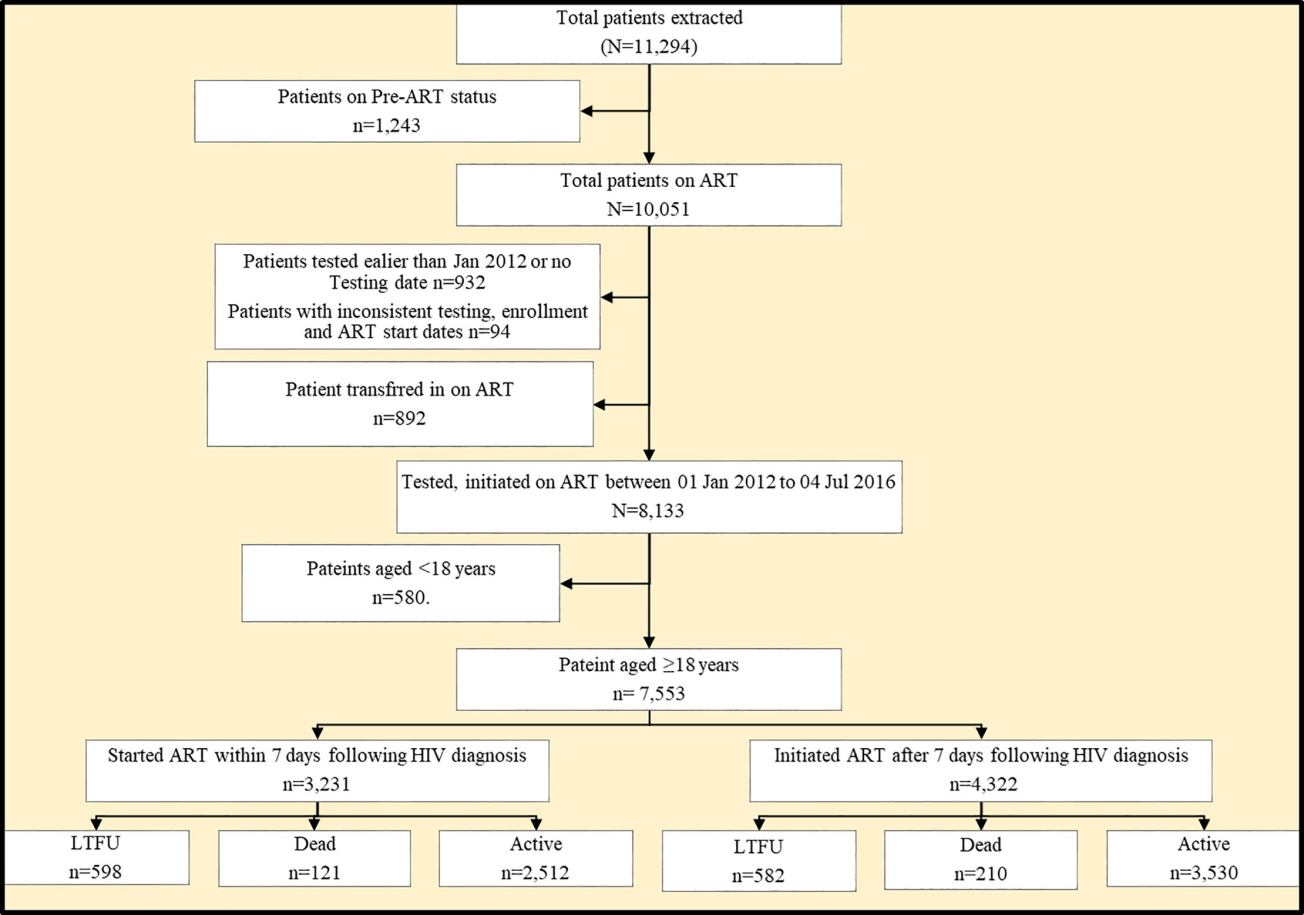
A flow diagram showing patients abstraction and inclusion into the study and their outcomes.

During the period 1^st^ January 2012 to 4^th^ July 2016, data for 11,294 patients were extracted and 7,553 patients met the study inclusion criteria (Fig 1). Overall, there were more females (62.3%) than males. The median (IQR) age at initiation of ART was 30 (25–37) years with 20% starting ART at 40 years or above. Majority of patients were married (41.5%), had attained at least primary education (53.8%), weighed 45-60kg (62.1%), did not have a telephone set (50.9%), had no signs of tuberculosis (TB) (88.9%), started ART in WHO clinical stage 1 or 2 (85.5%), started treatment with Tenofovir Disoproxil Fumarate (TDF) based regimen (76.0%), and had a baseline body mass index (BMI) of 18.51–29.99 (75.7%). The median (IQR) CD4 cell count was 327 (180–485) cells/ml. Details of the baseline patients’ characteristics are shown in Table 1.

**Table 1:** Patients background characteristics.

Characteristic	Categories					All Groups		
(s)		ART initiated within 7 days following diagnosis		ART initiated after 7 days following diagnosis				
		n	%	n	%	n	%	P-value
Sex	Male	1099	38.6	1747	40.4	2846	37.7	0.339
	Female	2132	66.0	2575	59.6	4707	62.3	<0.001
Age (years)	Median (IQR)	30 (25–37)		30 (25–38)		30 (25–37)		
	18–24	769	23.8	892	20.6	1661	22.0	0.117
	25–29	846	26.2	1064	24.6	1910	25.3	0.424
	30–39	987	30.6	1509	34.9	2496	33.1	0.026
	40–49	428	13.3	606	14.0	1034	13.7	0.747
	50+	201	6.2	251	5.8	452	6.0	0.859
Marital Status	Never Married	204	6.3	318	7.4	522	6.9	0.630
	Married	1447	44.8	1686	39.0	3133	41.5	0.001
	Divorced/Separated	630	19.5	930	21.5	1560	20.6	0.339
	Widowed	43	1.3	80	1.9	123	1.6	0.806
	Missing	907	28.1	1308	30.3	2215	29.3	0.264
Education	None	216	6.7	348	8.1	564	7.5	0.541
	Primary	1648	51.0	2416	55.9	4064	53.8	0.002
	Secondary	965	29.9	1143	26.5	2108	27.9	0.083
	Post-secondary	149	4.6	155	3.6	304	4.0	0.660
	Missing	253	7.8	260	6.0	513	6.8	0.421
Has Telephone	No	1337	41.4	2507	58.0	3844	50.9	<0.001
	Yes	1894	58.6	1815	42.0	3709	49.1	<0.001
Baseline Weight (Kgs)	Mean (SD)	55 (10.9)		55 (10.5)		55(10.7)		
	<45	310	9.7	453	10.5	763	10.2	0.720
	45–60	1963	61.6	2692	62.5	4655	62.1	0.532
	61+	916	28.7	1163	27.0	2079	27.7	0.390
Baseline BMI	≤18.50	376	21.9	605	21.2	981	21.5	0.795
	18.51–29.99	1293	75.3	2168	75.9	3461	75.7	0.691
	≥30	48	2.8	83	2.9	131	2.9	0.974
Baseline CD4 cell count (μ/ml)	Median (IQR)	300 (171–440)		348(190–520)		327 (180–485)		
	<200	932	30.9	1137	26.9	2069	28.5	0.045
	200–350	864	59.6	995	50.4	1859	25.7	<0.001
	≥351	1219	40.4	2100	49.6	3319	45.8	<0.001
Baseline WHO clinical stage	1&2	2836	88.1	3605	83.6	6441	85.5	<0.001
	3&4	383	11.9	710	16.5	1093	14.5	0.042
TB status	No signs	2961	91.6	3750	86.8	6711	88.9	<0.001
	TB Suspect	229	7.1	216	5.0	445	5.9	0.354
	TB Diagnosed	5	0.2	255	5.9	260	3.4	0.712
	TB treatment	18	0.6	90	2.1	108	1.4	0.666
	Missing	18	0.6	11	0.3	29	0.4	0.910
Baseline ART regimen	ABC based	14	0.4	10	0.2	24	0.3	0.932
	AZT based	468	14.5	1317	30.5	1785	23.6	<0.001
	TDF based	2747	85.0	2990	69.2	5737	76.0	<0.001
	Other	2	0.1	5	0.1	7	0.1	0.999
Year of Starting ART	2012	292	9.0	1056	24.4	1348	17.9	<0.001
	2013	538	16.7	1253	29.0	1791	23.7	<0.001
	2014	940	29.1	980	22.7	1920	25.4	0.001
	2015	993	30.7	782	18.1	1775	23.5	<0.001
	2016	468	14.5	251	5.8	719	9.5	0.001

### Study outcomes

#### a) Overall loss to follow up

**Fig 2:**
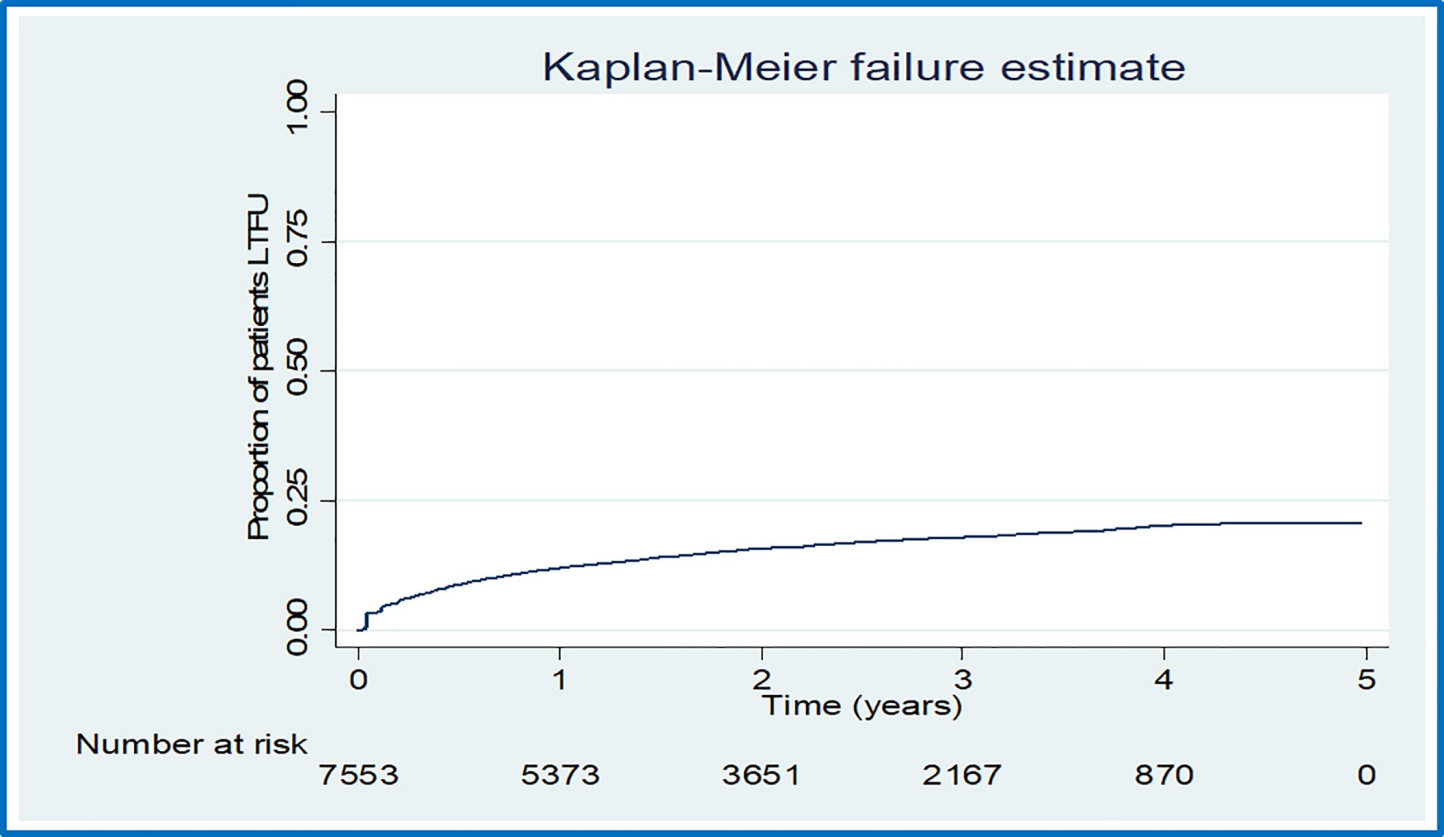
Overall proportion of patients lost to follow up in the study period.

**Fig 3:**
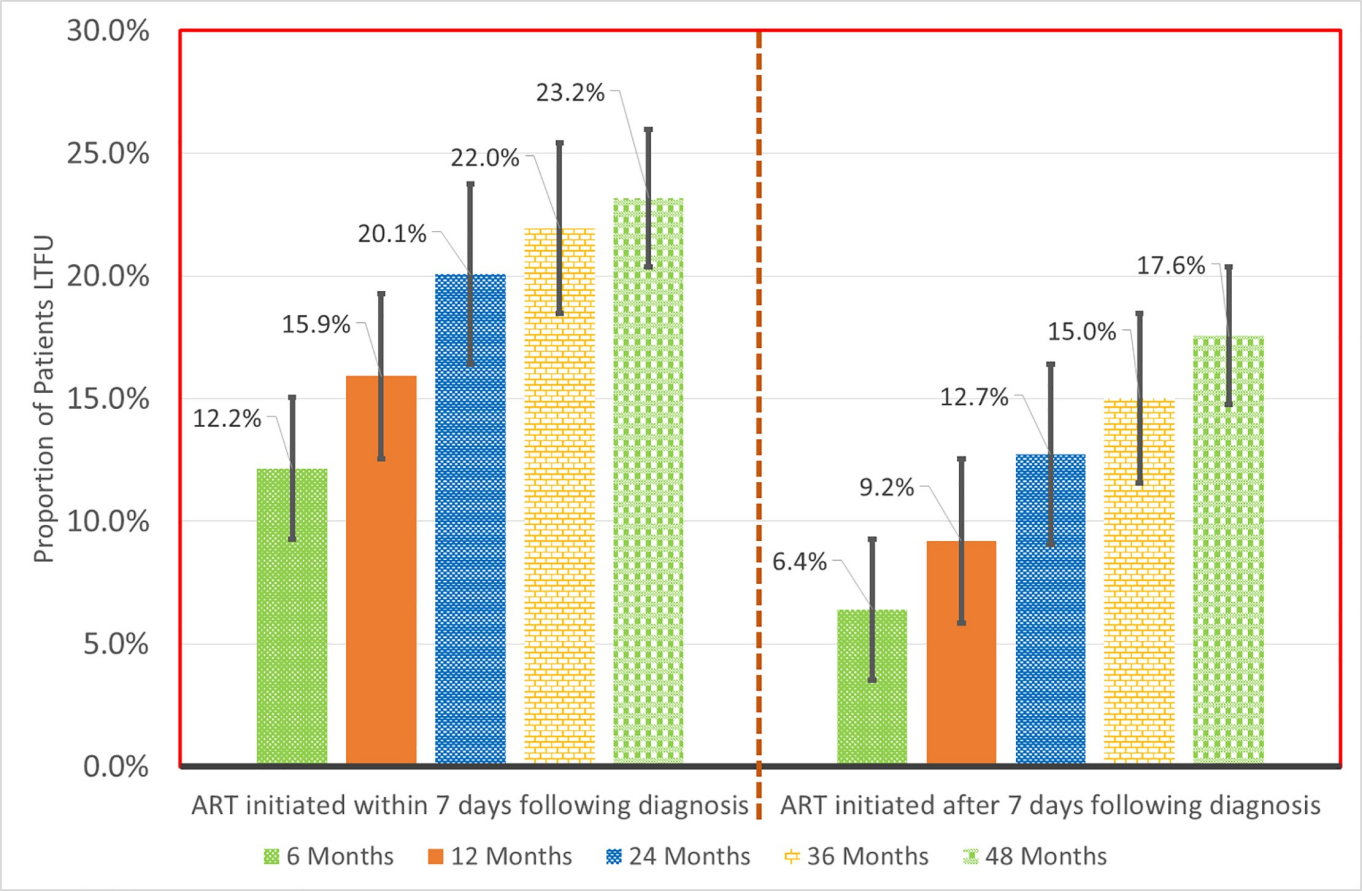
Cumulative incidence of loss to follow up by study group at different time points.

There were 1180 cases of LTFU observed over 15,807.7 person years at risk. The overall incidence rate (IR) of LTFU was 7.5 (95% CI = 7.1–7.9) per 100 person years of observation (pyo). Cumulative incidence of LTFU was 8.9% (95% CI = 8.2–9.6%) at 6 months, 12.1% (95% CI = 11.3–12.9%) at 12 months, 15.8% (95% CI = 15.0%-16.7%) at 24 months, 18.0% (95% CI = 17.0–19.0%) at 36 months and 20.2% (95% CI = 19.0–21.4%) at 48 months. Fig 2 shows the overall cumulative incidence of LTFU during the study period. Fig 3 illustrates the proportion of patients LTFU by study group.

#### b) Incidence rate of loss to follow up by patients characteristics

Table 2 depicts detailed incidence rates (IR) of LTFU by patients’ baseline characteristics, where the IR of LTFU was higher in the group that started ART within 7 days following diagnosis compared to the group that started ART after 7 days following diagnosis. The incidence rate of LTFU was highest in patients that started ART aged 18–24 years, highest in patients who were never married, highest in patients without a telephone set, highest in patients whose HIV disease was classified as either WHO stage 3 or 4 at baseline, highest among TB suspects at and highest among those whose baseline ART regimens were Abacavir (ABC) based.

**Table 2:** Incidence rate of loss to follow up by patients characteristics.

Characteristic(s)	Categories	Number of Cases	Person time	Rate/100PYO	95% CI	
Overall study group		1180	15807.7	7.5	7.1	7.9
Study Group	ART initiated within 7 days following diagnosis	598	5600.0	10.7	9.9	11.6
	ART initiated after 7 days following diagnosis	582	10207.8	5.7	5.3	6.2
Patient's Sex	Male	434	5943.5	7.3	6.6	8.0
	Female	746	9864.2	7.6	7.0	8.1
Age group (years)	18–24	419	2913.7	14.4	13.1	15.8
	25–29	306	3939.9	7.8	6.9	8.7
	30–39	312	5541.7	5.6	5.0	6.3
	40–49	99	2386.0	4.1	3.4	5.1
	50+	44	1026.4	4.3	3.2	5.8
Patient's Marital Status	Never Married	103	982.2	10.5	8.6	12.7
	Married	477	6554.2	7.3	6.7	8.0
	Divorced/Separated	261	3042.7	8.6	7.6	9.7
	Widowed	14	306.2	4.6	2.7	7.7
	Missing	325	4922.5	6.6	5.9	7.4
Highest Education level attained	None	110	1157.0	9.5	7.9	11.5
	Primary	641	8609.6	7.4	6.9	8.0
	Secondary	311	4356.0	7.1	6.4	8.0
	Post-secondary	30	675.3	4.4	3.1	6.4
	Missing	88	1009.9	8.7	7.1	10.7
Whether Patient has Telephone	No	701	8429.7	8.3	7.7	9.0
	Yes	479	7378.0	6.5	5.9	7.1
Baseline Weight (Kgs)	<45	128	1452.9	8.8	7.4	10.5
	45–60	770	9734.9	7.9	7.4	8.5
	61+	253	4590.7	5.5	4.9	6.2
Baseline BMI	≤18.50	141	1918.3	7.4	6.2	8.7
	18.51–29.99	467	7674.9	6.1	5.6	6.7
	≥30	5	368.0	1.4	0.6	3.3
Baseline CD4 cell count (μ/ml)	<200	254	4344.0	5.8	5.2	6.6
	200–350	276	4165.7	6.6	5.9	7.5
	≥351	457	7195.0	6.4	5.8	7.0
Baseline WHO clinical stage	1&2	998	13911.7	7.2	6.7	7.6
	3&4	173	1892.4	9.1	7.9	10.6
Patient's baseline TB status	No signs	1036	14437.1	7.2	6.8	7.6
	TB Suspect	74	759.8	9.7	7.8	12.2
	TB Diagnosed	36	411.2	8.8	6.3	12.1
	TB Treatment	12	197.7	6.1	3.4	10.7
Baseline ART regimen	ABC based	4	32.9	12.2	4.6	32.4
	AZT based	237	5378.2	4.4	3.9	5.0
	TDF based	938	10382.2	9.0	8.5	9.6
	Other	1	14.4	6.9	1.0	49.2

#### c) Factors associated with loss to follow up

Table 3 depicts the unadjusted and adjusted hazard rates of factors associated with LTFU. None of the variables included in the final model violated the PH assumption. At bivariate, patients who started ART within seven days following HIV diagnosis were 59% more likely to be lost to follow up (crude hazard ratio (cHR) = 1.59, 95% CI, 1.42–1.79).

**Table 3:** Factors associated with loss to follow up.

Characteristic (s)	Categories	crude Hazard Ratios	95% CI	P value	Adjusted Hazard Ratios	95% CI	P value
Study Group	ART initiated after 7 days following diagnosis	1.00			1.00		
	ART initiated within 7 days following diagnosis	1.59	1.42–1.79	<0.001	1.69	1.50–1.91	<0.001
Patient's Sex	Male	1.00					
	Female	1.04	0.93–1.17	0.503			
Age group (years)	18–24	1.00			1.00		
	25–29	0.58	0.50–0.67	<0.001	0.57	0.49–0.66	<0.001
	30–39	0.43	0.37–0.50	<0.001	0.41	0.35–0.48	<0.001
	40–49	0.32	0.26–0.40	<0.001	0.30	0.24–0.37	<0.001
	50+	0.33	0.24–0.45	<0.001	0.29	0.21–0.40	<0.001
Patient's Marital Status	Never Married	1.00			1.00		
	Married	0.76	0.61–0.94	0.012	0.95	0.76–1.18	0.656
	Divorced/Separated	0.84	0.68–1.04	0.104	1.09	0.87–1.36	0.446
	Widowed	0.55	0.33–0.92	0.023	0.97	0.57–1.64	0.896
Highest Education level attained	None	1.00			1.00		
	Primary	0.81	0.66–0.99	0.037	0.77	0.62–0.94	0.012
	Secondary	0.77	0.62–0.95	0.017	0.65	0.52–0.82	<0.001
	Post-Secondary	0.50	0.34–0.75	0.001	0.50	0.34–0.75	0.001
Whether Patient has Telephone	Yes	1.00			1.00		
	No	1.39	1.24–1.57	<0.001	1.52	1.35–1.71	<0.001
Baseline CD4 cell count (μ/ml)	<200	1.00			1.00		
	200–350	1.18	0.99–1.41	0.065	1.21	1.01–1.45	0.038
	≥351	1.10	0.95–1.28	0.202	1.13	0.96–1.32	0.133
Baseline WHO clinical stage	1&2	1.00			1.00		
	3&4	1.15	0.98–1.36	0.081	1.35	1.10–1.65	0.004
Patient's baseline TB status	No signs	1.00			1.00		
	TB Suspect	1.20	0.95–1.52	0.134	1.12	0.87–1.44	0.371
	TB Diagnosed	1.02	0.73–1.42	0.924	1.12	0.76–1.64	0.559
	TB Treatment	0.77	0.44–1.36	0.369	0.71	0.39–1.29	0.261
Baseline BMI	≤18.50	1.00			1.00		
	18.51–29.99	0.92	0.75–1.12	0.380	0.94	0.75–1.17	0.580
	≥30	0.50	0.31-.82	0.006	0.60	0.37–0.97	0.039

In the multivariable analysis, the risk of LTFU was 69% higher in patients who started ART within seven days following HIV diagnosis compared to those who began ART after seven days (adjusted hazard ratio (aHR) = 1.69, 95% CI, 1.50–1.91). Patients who started ART at age 18–24 years were more likely to be LTFU compared with all the other age groups (aHR = 0.57, 95% CI, 0.49–0.66 for patients aged 25–29 years, aHR = 0.41, 95% CI, 0.35–0.48 for patients aged 30–39 years, and aHR = 0.29, 95% CI, 0.21–0.40 for patients aged 50 years or more). Compared to patients with no education, the risk of LTFU was least among patients who started ART with post-secondary education level (aHR = 0.50; 95% CI, 0.34–0.75). Patients without a telephone set were 52% more likely to be LTFU compared to those with a telephone set (aHR = 1.52, 95% CI, 1.35–1.71). Patients who started ART with HIV disease classified as WHO stage 3 or 4 were 35% more likely to be LTFU compared to those with WHO stage 1 or 2 (aHR = 1.35, 95% CI, 1.10–1.65). Patients who started ART with a baseline CD4 cell count of 200–350μ/ml were 21% more likely to be LTFU compared to those with a baseline CD4 cell count of <200 μ/ml (aHR = 1.21, 95% CI, 1.01–1.45). Patients who started ART with a BMI of 30.0 or more, had a lower risk of LTFU compared to those who started ART with a BMI of <18.50 (aHR = 0.60, 95% CI, 0.37–0.97) There was no association between patients’ marital status and baseline TB assessment status with the risk of LTFU.

## Discussion

In this retrospective observation study in a primary healthcare clinic implementing the HIV T&T approach, we observed a higher cumulative incidence of LTFU in the entire study cohort. The LTFU rate was 7.5 per 100 years of observation. The likelihood of LTFU was higher among patients who started ART within seven days following an HIV diagnosis, were aged ≥25 years, did not have access to a telephone set, were less educated, had a baseline CD4 cell count of 200–350μ/ml and started ART with WHO stage categorized as either 3 or 4 or had a BMI of ≤18.50.

Four years after starting ART, one in every five patients (20.2%) who had started ART was LTFU. This falls short of the 2^nd^ UNAIDS target related to 90% of those diagnosed with HIV infection receiving sustained ART [2]. At 12 months after starting ART, we observed similar rates of loss to follow up as those reported by studies under the universal T&T setting [23,17], and in other non T&T settings [24,25]. Despite differences in the methodology and outcome definitions, we consistently observed an increasing cumulative incidence of LTFU with increase in years of treatment, similar to another study in South Africa [26]. To enhance long-term patient retention therefore, HIV programs must initiate concerted efforts that target the first months of treatment in ART-naïve patients. Besides, our findings also show an elevated risk of LTFU among patients who started ART within seven days following diagnosis compared to those who started after 7 days following HIV diagnosis. This finding contrasts what has been reported previously [27]. One possibility for the differences could be methodological, especially in the determination of the outcomes across the studies, but also the fact that different study populations were considered. Within the seven days, it is unconvincing that patients would have received enough counselling and consequently would have appreciated the benefit of starting ART when they were not yet “feeling sick” at diagnosis. Addressing structural bottle necks including counseling after a positive HIV test and before ART initiation have been identified as strategies for improving ART adherence and retention [28]. Before the T&T approach, the emphasis was that patients were taken through a minimum of three counselling sessions, were required to bring a treatment supporter, and had to demonstrate understanding of long term treatment by answering questions after counselling [29]. Currently however, ART is only delayed when there are co-infections with TB or Cryptococcal meningitis. All other patients’ psycho-socio concerns are addressed along the treatment cascade during the subsequent clinic visits. It is therefore possible that some patients start ART when they have not fully accepted the benefits of early ART and the need to adhere to subsequent clinic visits. Therefore, in this T&T era, we suggest that ART programs initiate, and maintain an enhanced therapeutic education program throughout the HIV cascade to improve retention.

Between 2004 and 2012, ART was not commonly provided in health centers at level III (Health Centers at level three) [30]. In the Ugandan setting, these provide outpatient, maternity, general ward and laboratory services. However, at the beginning of 2013 and beyond, most health centers at level III were ART accredited. This is suggestive that a large number of patients formerly at Uganda Cares clinic might have opted to receive ART services at these centers without formally seeking transfers/referrals. Self-transfers across ART programs have been illustrated to conceal the actual proportion of patients categorized as LTFU in an Ethiopian study in a setting similar to ours [31]. The Uganda Cares clinic serves as the main HIV OPD clinic for the Masaka regional referral hospital, and therefore there is a possibility that patients diagnosed HIV positive on wards and started ART immediately or within 7 days, opted to receive ART at health facilities nearest to their usual dwellings once they got better. Furthermore, these patients could have died after starting ART but were never reported given the passive nature of surveillance in our setting. Under the Option B+ of PMTCT program, where immediate ART initiation has widely been practiced, patients’ retention in such settings has remained sub optimal. And, initiation of ART on the same day of testing HIV positive was independently associated with an elevated risk of loss to follow up in the initial months of starting ART [15,32].

Similar to a study done in Kenya [19], retention rates in all other age groups were better compared to adolescents or young adults (18–24 years). Retention in adolescents and young adults should be an important subject given the rising rates of infection in this particular sub population [33] and high rates of viral un-suppression [34]. If not at school, adolescents and young adults are usually at conflict with work schedules and most times fail to make routine monthly schedules, a requirement in most ART clinics. Similarly, clumping ART services of adults together with those of adolescents and young adults might blur individualized adolescent and young adults’ needs. We anticipate that adolescents and young adults’ groups might benefit from differentiated care models that address individualized patients’ needs.

As seen from another study [35], patients with no education were more likely to be LTFU. Such patients might find it hard to read text reminder messages plus a couple of other information education communication (IEC) materials in health facilities that stress the need for continued care.

We observed that clients starting ART in WHO clinical stage 3 or 4 were more likely to be LTFU compared to those in stage 1 or 2. Among asymptomatic patients who started ART in South Africa, the proportion of LTFU was low [24]. Additionally, a reduced risk of death and improved retention rates were observed in a study assessing effectiveness of a streamlined model of care [23]. Patients categorized as stage 3 or 4 show higher likelihood of infections and are bed ridden most of the time. This is likely to make their continued engagement with the HIV clinic difficult. Such patients also have an increased risk of death especially in the first 6 months of ART due to severe immune reconstitution inflammatory syndrome and Cryptococcal meningitis [36,37]. It is therefore possible that they died shortly after starting ART and never reported to the ART clinic again.

We observed that patients with a telephone set (mostly mobile phone) were less likely to be LTFU. In our setting, patients are sent short reminder text messages (SMS) before the clinic day and those who miss a clinic day are immediately called for a re-appointment. This cannot happen if one has no phone and so may lead to loss to follow up. Mobile phone technologies (mHealth), specifically SMS reminders have improved patient outcomes in other health service delivery settings [38–40] but were comparable to the standard of care for HIV retention in other settings [41,22,42]. It is however important to note that the level of interaction between provider and patient, and subset of activities under mHealth greatly determine the effectiveness of particular interventions. Therefore, interactive SMS reminders alone might not improve patient outcomes when compared to a combined strategy of SMS reminders, home visits and direct phone calls to patients.

Our observed association between baseline CD4 cell count and LTFU was consistent with findings from studies by Jain et al and Hønge et al [24,43], despite differences in methodologies. We found a reduced likelihood of being LTFU among patients with high CD4 cell count (i.e, ≥ 351μ/ml). However, in a study by Mberi and colleagues [26], there was an elevated risk of LTFU among patients with a baseline CD4 cell count of >200μ/ml. One possibility for this contrast could be a result of differences in the classification of a patient as LTFU but also immunological differences among study patients. Mberi et al classified patients as LTFU if they had spent at least 180 days without ART from the HIV clinic after a scheduled visit and this is different from our definition of LTFU. Similarly, our median (IQR) baseline CD4 cell count was high (327 (180–485)) compared to the best group in the study by Mberi et al of 87(36–150) [26]. Besides, patients with low CD4 cell counts are likely to be in WHO stage 3 or 4, a group prone to opportunistic infections and an elevated risk of mortality [36,37]. It is therefore possible that patients with lower CD4 cell counts in our study were bed ridden or died shortly after initiation of ART and were never reported to the HIV clinic.

The effect of baseline BMI on LTFU in our study is similar to findings from two retrospective studies done in Guinea-Bissau and Malawi. Both studies established that a BMI of <18.5kg/m^2^ was associated with an increased risk of LTFU [43,44]. The low risk of LTFU we observed in over-weight patients could have arisen out of the enhanced counseling support offered in the non-communicable diseases (NCDs) clinic that was established during the course of the study. Health education messages in this clinic were tailored towards improving drug adherence as well as requiring patients to routinely visit the health facility for routine monitoring; and the clinic targeted patients with a higher risk of developing hypertension and diabetes. It is also possible that patients who were under-weight, developed opportunistic infections related to malnutrition and later died but were never reported to the HIV clinic due to the passive reporting mechanism. Moreover, a lower BMI has been reported to be correlated with HIV mortality and progression to AIDS [45,46].

### Limitations

Our study had limitations that should be taken into account while interpreting these findings. First, we utilized already collected data used for routine patient management. Such data presents with lots of gaps and sometimes may not present the rigor to warrant scientific research. Whereas this particular clinic is not a research site, it is part of the east African International epidemiology database to evaluate AIDS (IeDEA) consortium. As such, there are inherent data validation rules within the database and daily data cleaning to guarantee a certain degree of data correction and collation. Secondly, as it is in most HIV programs, there is a passive nature of surveillance and follow up of patients. There is therefore a possibility of having determined and regarded patients as lost to follow up in Masaka when they are actually in HIV care and receiving treatment somewhere else. This therefore, might have resulted in some over-estimation of the cumulative incidence rates of LTFU in our study. There is however a dedicated team at the facility that does contact tracing/case navigation for clients who miss clinic appointments, and we believe this might have minimized the misclassification. Lastly, the nature of data collected was limited. Some of the many health system (human resource, waiting time, distance) and socio-economic (type of work, socio contacts, HIV disclosure) factors known to affect LTFU were not studied. The effect of such under the current study settings remained unknown. We anticipate that, examining the effect of these under a T&T setting could better inform ART programing and policies towards boosting retention. Despite this study’s limitations, the findings are likely to be generalizable to HIV positive patients who start ART in Uganda and in other resource-limited settings. Our study cohort was derived from a regional referral HIV clinic, therefore, it is highly possible that a wide scope of HIV patients with different background characteristics were studied. Furthermore, the HIV clinic to the largest extent exercises passive surveillance, a practice evident in most HIV care programs in Uganda.

## Conclusions

Our study showed that the risk of loss to follow up increased with time and was higher among patients who start ART within seven days following HIV diagnosis, higher among patients who lack access to a telephone, lower among patients aged ≥ 25 years, lower among patients with at least primary education, and lower among patients with BMI of ≥ 30. In this T&T era, there is need for HIV programs to initiate and maintain an enhanced therapeutic education program that target high risk groups, as well as leveraging on mHealth to improve patients’ retention into care throughout the entire cascade of HIV care.

## Supporting information

S1 Dataset (XLSX)
